# Overexpression of Three *TaEXPA1* Homoeologous Genes with Distinct Expression Divergence in Hexaploid Wheat Exhibit Functional Retention in *Arabidopsis*

**DOI:** 10.1371/journal.pone.0063667

**Published:** 2013-05-16

**Authors:** Zhaorong Hu, Na Song, Jiewen Xing, Yanhong Chen, Zongfu Han, Yingyin Yao, Huiru Peng, Zhongfu Ni, Qixin Sun

**Affiliations:** 1 State Key Laboratory for Agrobiotechnology, Key Laboratory of Crop Heterosis, Utilization (MOE), Beijing Key Laboratory of Crop Genetic Improvement, China Agricultural University, Beijing, People’s Republic of China; 2 National Plant Gene Research Centre, Beijing, People’s Republic of China; East Carolina University, United States of America

## Abstract

Common wheat is a hexaploid species with most of the genes present as triplicate homoeologs. Expression divergences of homoeologs are frequently observed in wheat as well as in other polyploid plants. However, little is known about functional variances among homologous genes arising from polyploidy. Expansins play diverse roles in plant developmental processes related to the action of cell wall loosening. Expression of the three *TaEXPA1* homoeologs varied dynamically at different stages and organs, and epigenetic modifications contribute to the expression divergence of three *TaEXPA1* homoeologs during wheat development. Nevertheless, their functions remain to be clarified. We found that over expression of *TaEXPA1-A*, *-B* and *-D* produced similar morphological changes in transgenic *Arabidopsis* plants, including increased germination and growth rate during seedling and adult stages, indicating that the proteins encoded by these three wheat *TaEXPA1* homoeologs have similar (or conserved) functions in *Arabidopsis*. Collectively, our present study provided an example of a set of homoeologous genes expression divergence in different developmental stages and organs in hexaploid wheat but functional retention in transgenic *Arabidopsis* plants.

## Introduction

Polyploidization plays an important role in plant evolution. It has been demonstrated that approximately 70% of flowering plants experienced polyploidization events during their evolution [1], [2]. Polyploidy, along with genomic segmental duplications, could benefit plants by increasing overall gene expression levels and cell sizes, and providing sources for novel variants and genome “buffering” of deleterious mutations [3]. When two or more different genomes are combined into a single cell, they must respond to the consequences of genome duplication [2], [4], [5], [6]. There are three possible evolutionary fates for homoeologous genes in polyploids: retention of original or similar function, functional diversification, and gene silencing [2].

Hexaploid bread wheat (*T. aestivum*, 2n = 6x = 42, genome constitution AABBDD) is an allopolyploid that was formed through hybridization and successive chromosome doubling of three ancestral diploid species (2n = 14), *T. urartu* (AA), *Aegilops speltoides* (SS ≈ BB), and *Ae. squarrosa* (DD) [7], [8]. Allopolyploidization leads to the generation of duplicated homoeologous genes (homoeologs). Therefore, most genes in hexaploid wheat are present as triplicate homoeologs. However, the presence of three homoeologs in wheat does not necessarily imply that three independent mRNAs are transcribed, and transcriptional divergence between homoeologous genes is widely documented in hexaploid wheat [9], [10], [11], [12], [13], [14]. Organ-specific regulation of homoeologous gene expression is also frequently observed [9]. Although there are numerous studies analyzing the genomic structures and transcriptional divergences of the homoeologous genes in hexaploid wheat, little is known about functional variances among homologous genes arising from polyploidy in wheat. Such information, however, is fundamentally important for a better understanding of the complex mechanisms involved in variant biological pathways in the hexaploid wheat and for the benefit of the genetic engineering of plants with polyploid backgrounds. For example, systematic study revealed that homoeologous *Bx* genes (*TaBx1–TaBx5*) were highly conserved in the coding sequences and in the exon/intron structures, but were transcribed differentially in which the homoeologs from the B genome generally contributed the most to Bx biosynthesis in hexaploid wheat [15]. Recently, Abdollahi et al (2012) reported that *WAP2A^Q^* fully recovered their flower organs similar to the wild-type, *WAP2D* showed less recovery, and *WAP2A^q^* rescued the least mutant flower phenotype [16].

Expansins are a family of closely related nonenzymatic proteins that induce cell wall extension and stress relaxation at acidic pH condition in plants [17]. Based on phylogenetic analysis and shared intron patterns, expansins can be mainly divided into three discrete subfamilies, *α-*, *β* and *γ*-expansins [18], [19], [20]. They are similar to each other in size and have distant but significant sequence similarity throughout the length of the protein backbone. A number of sequence features are common to the three expansin families, including a signal peptide in the N-terminal region, a cellulose-binding domain in the C-terminal region, and a central catalytic domain that contains a histidine-phenylalanin-aspartate (HFD) motif and several cysteine residues thought to function in catalytic activity [21]. It has been shown that expansins play diverse roles in plant developmental processes related to the action of cell wall loosening [22], [23], [24], [25], [26].

In previous study, we identified three *TaEXPA1* homoeologous genes (*TaEXPA1-A*, *TaEXPA1-B* and *TaEXPA1-D*) from hexaploid wheat. Chromosome mapping using aneuploid lines revealed that the *TaEXPA1-A*, *TaEXPA1-B* and *TaEXPA1-D* genes located on chromosomes, 1A, 1B, and 1D, respectively. The orthologs of *TaEXPA1* also were found in the three ancestral diploid species. We demonstrated that epigenetic modifications contribute to the expression divergence of three *TaEXPA1* homoeologs during wheat development [27]. Due to its highly conserved sequence but transcriptional divergences in different organs and tissues, it is of great interest to reveal functional variances among three *TaEXPA1* homologous genes in wheat. In present study, we found that over expression of *TaEXPA1-A*, *-B* and *-D* produced similar morphological changes in transgenic *Arabidopsis* plants, including increased germination and growth rate during seedling and adult stages, indicating that the proteins encoded by these three wheat *TaEXPA1* homoeologs have similar (or conserved) functions in *Arabidopsis*.

## Materials and Methods

### Plant Materials

The hexaploid wheat genotype Nongda 3338 (*Triticum aestivum*) was used in this study. Wheat seeds were germinated on moist filter papers and transplanted into pots to grow in the greenhouse under a 12-hr photoperiod provided by cool white fluorescent and incandescent light (intensity≥3000 lx). The plants were watered with a 1/10-strength Hoagland’s solution when necessary. The Columbia (Col-0) ecotype of *Arabidopsis thaliana* was used as the wild type. All genotype plants were grown in a growth room with a 16-h light/8-h-dark cycle at 22 to 24°C for general growth and seed harvesting.

### Phylogenetic Analysis of *α*-expansin Genes

All deduced amino acid sequences were aligned using Clustal method with the MEGA4.0 program [28]. Distance analyses used the program ProtDist of the Phylip 3.5c package with a PAM250 substitution matrix. Phylogenetic trees were then calculated from the matrices by the neighbor-joining algorithm. Bootstrap analyses consisted of 1,000 to 5,000 replicates using the same protocol. The dendrogram was generated on the basis of the alignment of the deduced amino-acid sequences encoded by 26 *α*-expansin genes of *Arabidopsis*, 33 *α*-expansin genes of rice (http://www.personal.psu.edu/fsl/ExpCentral/), and 3 *TaEXPA1* homoeologs of wheat. The GenBank accession numbers of selected expansins can be found in the Table S1.

### RNA Extraction

Wheat Nongda 3338 were planted in pots in silt-loam/coarse soil mix and grown in green house. In two-leaf stage, the first leaf in the bottom, the second fully expanded leaf in the middle and the third unexpanded leaf were collected, and designated as mature, fully expanded and juvenile leaves, respectively. Total RNA extracted from different leaves for the gene expression analysis, using Trizol RNA isolation protocol (Life Technologies, USA). First-strand cDNA synthesis was performed using 2 µg of DNase-digested total RNA with oligo (dT) primer according to the protocol for RT-PCR first-strand synthesis (Promega, WI, USA).

### Quantitative PCR Analysis

Real-time qPCR experiments using SYBR Green PCR master mix (Applied Biosystems) on 96-well optical reaction plates with optical adhesive covers (ABI PRISM) were designed, performed, and analyzed using the StepOnePlus Real-Time PCR system with its accompanying StepOne Software (version 2.2; Applied Biosystems). Gene-specific primers for quantitative PCR were designed based on nucleotide polymorphisms in the cDNA sequences of three *TaEXPA1* homoeologous genes, Table S2). The specificity of the primers was confirmed using plasmids containing genomic DNA sequences of *TaEXPA1* homoeologs. The PCR efficiencies of each primer pair were determined from standard curve experiments. Any amplification with detected primer dimers from melt-curve analysis was excluded from the data analysis [29]. In comparative cycle threshold experiments for quantitative analysis of gene expression levels, *β-actin* was amplified as an endogenous control.

### Binary Constructs and *Arabidopsis* Transformation

To examine heterologous expression of *TaEXPA1* homoeologous genes, genomic DNA fragments of the genes were cloned downstream of the cauliflower mosaic virus 35S promoter in pB2GW7 vectors. Binary vectors were introduced into *Agrobacterium tumefaciens* GV3101. Six-week-old *Arabidopsis* plants were transformed via *Agrobacterium*-mediated transformation by the floral-dip method [30]. Transgenic plants were selected on Murashige and Skoog medium containing herbicides. The herbicides-resistant seedlings were transferred to soil. T3 generation transgenic plants were used for further experiments.

### Germination Assays

Seed lots to be compared were harvested on the same day from individual plants grown in identical environmental conditions. Seeds of each genotype were sterilized and cold treated for 3 d in the dark, then sown in triplicate (55–65 seeds from one individual plant a quarter of Petri dish) on MS medium. The plates incubated in a climate-controlled room (22°C, 16-h light/8-h-dark cycle). Germination was scored after 16 h imbibitions, we used radicle tip break through the seed coat as the seed germination standard. The number of geminating seeds was counted and converted into germination rate per 8 hour intervals for both transgenic lines and wild type plants. The average germination percentages ± SE of triplicates were calculated.

### Image Processing and Measurements

For leaf size measurement and analysis of Col-0 wild-type and transgenic plants, cotyledons and sixth leaves were harvested and measured digitally on photographs taken using a digital camera (Nikon D90) with a standard 15-cm ruler for scaling on a black background. Acquired photographs were processed using Photoshop (Adobe version CS5) with manual tracing of leaf outlines to generate leaf silhouettes. Based on these measurements, leaf area was calculated.

### Fresh Weight and Height Measurement

All genotype plants were grown in a growth chamber with a 16-h light/8-h-dark cycle at 22°C. Plants were grown for 3 weeks after sowing and were measured. The actual times were 22, 25 and 29 days after sowing (DAS). The fresh rosette was cut with a pair of scissors just above ground and for weight determination with electronic balance and calculation of the amount of green biomass. Plant height was measured from the base of the plant (aerial parts) to the tip using a cotton thread. The actual times were 29, 32 and 35 DAS. Each line 15 plants were selected for fresh weight and height analysis.

## Results

### Structure and Phylogenetic Analysis of *TaEXPA1* Genes

The alignment of genomic and their corresponding cDNA sequences of three *TaEXPA1* homeologous genes showed a significant conservation of the intron/exon organization within the open reading frame (ORF). The structures of three *TaEXPA1* homeologous genes were also confirmed by the identification of potential exon/intron junction sequences, which were consistent with the universal rule GT.AG. The ORF sequences of three *TaEXPA1* genes showed single nucleotide substitutions only, whereas the introns had more frequent base substitutions and insertions/deletions (Figure S1).

**Figure 1:**
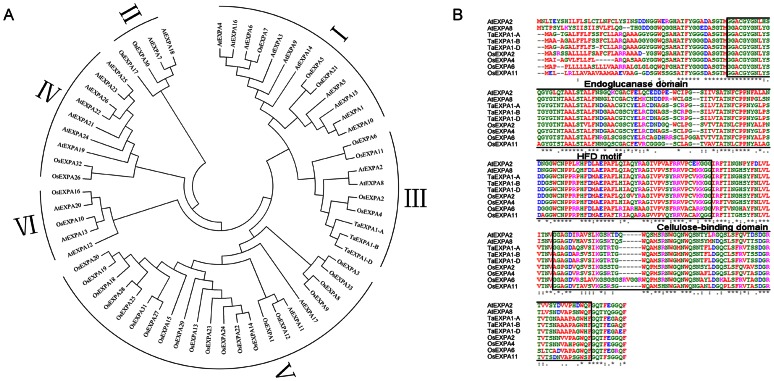
Sequence analysis of three *TaEXPA1* homoeologous genes. (A) Phylogenetic relationship of three TaEXPA1 homoeologs with *Arabidopsis* and rice *α*-expansins. The accession numbers of selected expansins are listed as Table S1. (B) Comparison of nine *α*-expansins amino acids in class III. Identical amino acids are indicated by asterisks, and black boxes indicate residues conserved among the seven proteins.

Based our previous results, the TaEXPA1 belongs to the *α*-expansin class [31]. To further investigate the relationship between three *TaEXPA1* homeologous genes and *α-*expansins of *Arabidopsis* and rice, we performed a neighbor-joining phylogenetic analysis using MEGA 4.0. These proteins were classified into six different classes according to the phylogenetic analysis, among which TaEXPA1 belongs to the class III (Figure 1A). Thus, these plant expansins proteins of class III were aligned using ClustalW, and the results showed that our identified wheat EXPA1 sequences contain highly conserved cellulose-binding domain, and a central catalytic domain that contains a histidine-phenylalanine-aspartate (HFD) motif and several cysteine residues thought to function in catalytic activity regions, which are thought to be critical for inducing cell wall extension and stress relaxation (Figure 1B).

It should be noted that, there were two and four expansin protein members that can be clustered into class III in *Arabidopsis* and rice, respectively (Figure 1A). Therefore, we speculate that there was at least another member that was not cloned in wheat.

### Expression Analysis of Three *TaEXPA1* Homoeologous Genes

**Figure 2:**
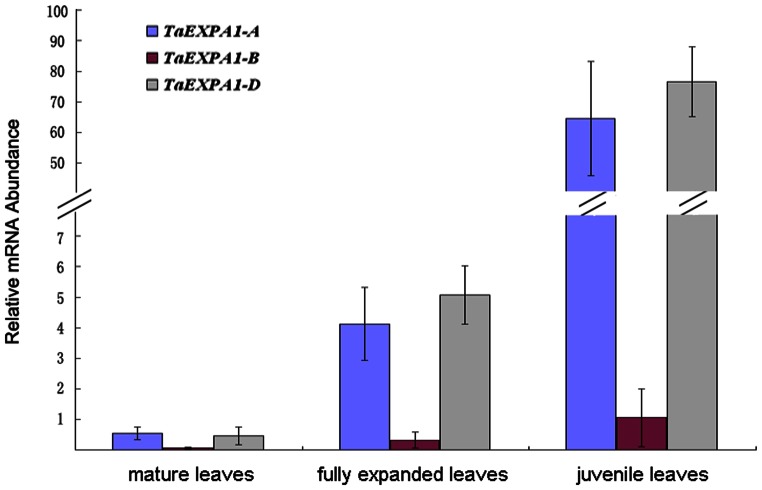
Expression patterns of *EXPA1* homoeologous genes by gene-specific PCR. Total RNA was isolated from different leaves of wheat genotype nongda 3338 in the two-leaf stage; *β-actin* was used as an endogenous control.

Previously, we examined the expression profiles of *TaEXPA1* homoeologs in different organs at different developmental stages. The results indicated that all three *TaEXPA1* homoeologous genes were expressed in dry seed embryos, shoots of 1d after germination seedlings, jointing stage stems, heading stage flag leaves and heading stage immature ears. However, expression levels of the three *TaEXPA1* homoeologs were significantly divergent in different tissues and different development stages. For example, all of the three *TaEXPA1* homoeologs were silenced in seedling roots. In the leaves of the same stage, *TaEXPA1-A* and *TaEXPA1-D* were expressed, but *TaEXPA1-B* was silenced [27]. Due to its important role in regulating cell wall extension during growth, here we examined the expression of three *TaEXPA1* homoeologs in different leaves of the two-leaf stage (Figure 2). Interestingly, expression divergence was observed between the three *TaEXPA1* homoeologs in seedling leaves of different development stages. Most notably, *TaEXPA1-A* and *TaEXPA1-D* homoeologs were with higher expression in the third juvenile leaves than the other expanded leaves, whereas the mRNA of *TaEXPA1-B* was below detection level (Figure 2).

### Overexpression of Three *TaEXPA1* Homoeologous Genes Displayed Increased Germination Rate in *Arabidopsis*

**Figure 3:**
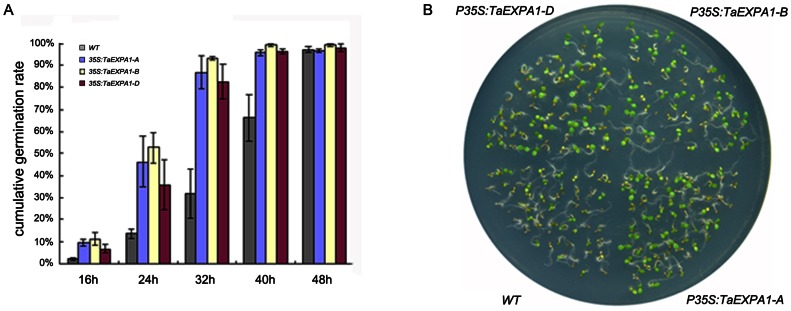
Transgenic *Arabidopsis* showed faster germination rates. (A) Germination was scored after 16 h imbibitions. The average germination percentages ± SE of triplicates were calculated. (B) *Arabidopsis* seeds germinated on MS medium after 90 h.

**Figure 4:**
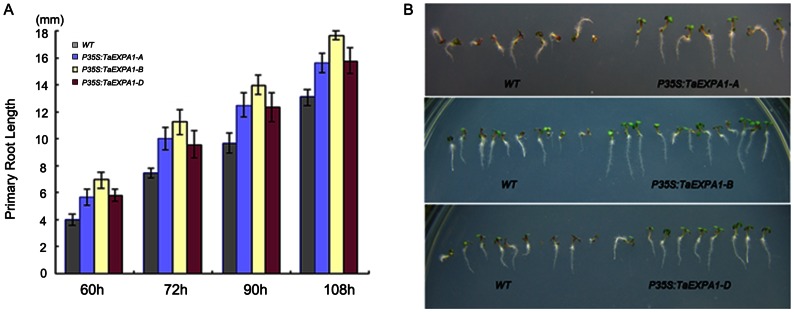
Transgenic *Arabidopsis* showed longer primary roots. (A) The primary root length of the transgenic lines and the wild-type plants were measured after 60 h at four time points. (B) Transgenic plants and wild-type sowed in MS medium after 72 h.

In order to gain further insight into the functional divergence of three *TaEXPA1* homoeologs genes in wheat, ectopic expression of *TaEXPA1-A*, *TaEXPA1-B* and *TaEXPA1-D* were performed using an *Arabidopsis* transgenic system. For every transgenic line, three independent T3 homozygous overexpression lines were used for further characterization. RT-PCR analysis indicated that three *TaEXPA1* homoeologs were expressed in all the transgenic *Arabidopsis* plants, respectively, but was absent in the wild-type (Figure S2). Overt phenotype was observed in transgenic *Arabidopsis* plants compared with control wild-type plants, and the transgenic plants with three *TaEXPA1* homoeologs genes constructs exhibited similar phenotypes. Firstly, we measured the germination rate between transgenic plants and wild-type plants. The number of geminating seeds was counted and converted into germination rate at 8 hour intervals for both transgenic lines and wild-type plants. The germination rate for transgenic lines were more than 40% in the first 24 hours after sowing, but only less than 20% for wild-type during the same time period. The cumulative germination rate of transgenic plants was more than 80%, where in contrast with only 30% for wild-type after 32 hours after sowing (Figure 3A). The phenotype differences were also observed in other independent transgenic lines, these data indicated that the transgenic lines germinated faster than the wild-type (Figure 3A, 3B). We also measured the primary root length of both transgenic and wild-type plants at 60, 72, 90 and 108 hours after sowing. At all four time points, primary root lengths of transgenic plants were longer than that of wild-type plants. This indicated that primary roots of transgenic plants grew faster than the wild-type (Figure 4).

### Overexpression of Three *TaEXPA1* Homoeologous Genes Lines Showed Larger Cotyledons and Rosette Leaves in *Arabidopsis*

**Figure 5:**
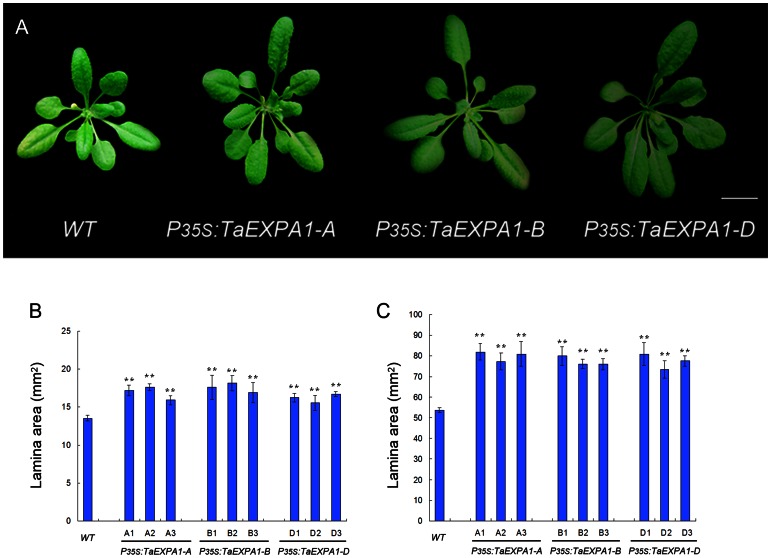
Transgenic *Arabidopsis* plants leads to an increase in rosette growth. (A) Transgenic *Arabidopsis* showed larger cotyledons and rosette leaves, Bar = 1cm. (B) Data show a comparison of lamina size between cotyledons from overexpression transgenic *Arabidopsis* plants and wild-type plants and measured at 30 DAS. Values are means±SE (n = 10). (C) Data show a comparison of lamina size between sixth leaves from overexpression transgenic *Arabidopsis* plants and wild-type plants and measured at 30 DAS. Values are means±SE (n = 10). T-test compared with the wild type control: *P<0.05, **P<0.01.

**Figure 6:**
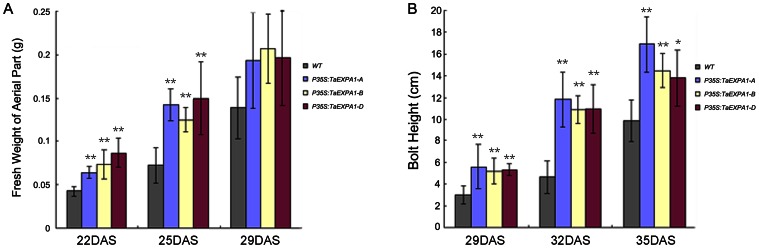
Transgenic *Arabidopsis* grow faster than non-transgenic seedlings. (A) Change of fresh weight of aerial tissues with time (DAS) between transgenic seedlings and wild-type plants. Values are means±SE (n = 15). T-test compared with the wild type control: *P<0.05, **P<0.01. (B) Change of bolt height with time (DAS) between transgenic seedlings and wild-type plants. Values are means±SE (n = 15). T-test compared with the wild type control: *P<0.05, **P<0.01.

Due to the divergent expression pattern of three *TaEXPA1* homoeologs genes in leaves, we focused on leaf growth phenotype between transgenic plants and wild-type plants. In comparison with control wild-type plants, transgenic lines of three *TaEXPA1* homoeologous genes displayed larger cotyledons (Figure 5A, B). Previous study showed that decreased expansin gene expression leads to a more marked repression of the leaf 6 growth during the later stage of leaf development [32]. To investigate the individual leaf basis of the changes after the overexpression of three *TaEXPA1* homoeologs, we analyzed the lamina area of leaf 6 at maturity (30 DAS) of the transgenic *Arabidopsis* plants with that of wild-type plants. These results showed that leaf lamina areas were all significantly increased in the overexpression of three *TaEXPA1* homoeologous genes plants (Figure 5A, C). Organ size is determined by final cell size and cell numbers. Previous studies indicated that repression of expansin gene expression leads to decreased cell number in both lamina and petiole [32]. To quantify this trait, we measured abaxial epidermal cell size of fully expanded sixth leaf (30 DAS) for both transgenic and wild-type plants using an inverted microscope. Cytological observation showed that epidermal cell size of transgenic and wild-type plants did not differ significantly (Figure S3), while the transgenic plants rosette leaves was significantly larger than that of wild-type (Figure 5A, C), implying that larger transgenic plants are mainly caused by increased cell numbers. Biomass is usually used as an index of plant growth rate. We then measured the fresh weight of aerial tissues in transgenic seedlings and wild-type plants at 22 days after sowing. The three *P35S:TaEXPA1* homoeolog transformants appeared heavier of fresh weight than non-transgenic seedlings at 22 and 25 DAS (Figure 6A). In addition, *Arabidopsis* plants transformed with *P35S:TaEXPA1* exhibited a taller phenotype than the wild-type plants (Figure 6B).

## Discussion

### Sequence Conservation, Expression Divergence and Functional Retention of Three *TaEXPA1* Homoeologous Genes

Previous classifications of the expansin gene family divided proteins into two subfamilies, *α* and *β*, based on substrate specificity and sequence conservation [21]. Further studies revealed a total of 38 expansin or expansin-like sequences within the scope of whole genome in *Arabidopsis*, these expansins fall into three discrete subfamilies (*α-*, *β* and *γ*) based on overall sequence similarity and shared structural features [20]. In previous work, we identified three *TaEXPA1* homoeologous genes *TaEXPA1-A*, *TaEXPA1-B* and *TaEXPA1-D* from hexaploid wheat [27]. Here, the relationship between three *TaEXPA1* homeologous genes and *α-*expansins of *Arabidopsis* and rice was further investigated by neighbor-joining phylogenetic analysis. These *α*-expansins were classified into six different classes according to the phylogenetic tree, among which three *TaEXPA1* homoeologs belong to the class III with *AtEXPA2*, *AtEXPA8* and *OsEXPA2*, *OsEXPA4*, *OsEXPA6*, *OsEXPA11* (Figure 1A), suggested functional similar between them. Based on an analysis with the ClustalW program, it was found that three TaEXPA1 homoeologs expansin proteins had a cleavable signal peptide at the N-terminus. Two domains characteristic of expansin proteins, a cysteine-rich region and a tryptophan-rich C-terminal portion, were also observed in all three *TaEXPA1* homoeologs, implied they may have conserved function.

Up to date, many wheat expansin genes were identified and their expression were analyzed. For example, 18 expansin genes were isolated from wheat and nine of them (*TaEXPA1–TaEXPA9*) represent *α*-expansins, while *TaEXPB1–TaEXPB5* and *TaEXPB7–TaEXPB10* belong to the *β*-expansin class [31]. Jin et al (2006) identified two wheat expansin genes that are expressed during male gametophyte development [33]. Gao et al (2008) evaluated the characteristics of wheat expansins protein TaEXPB23, and found the growth of wheat coleoptiles is intimately correlated with its expression [34]. In our previous report, it was found that all the three *TaEXPA1* homoeologs were silenced in seedling roots, but *TaEXPA1-A* and *-D* were expressed in seedling leaves [27]. In present study, expression pattern of these three *TaEXPA1* homoeologs was further investigated in seedling leaves of different development stages. Interestingly, *TaEXPA1-A and TaEXPA1-D* homoeologs appeared to be highly expressed in the young leaves, as compare to fully expanded leaves. In *Arabidopsis*, it was reported that expansin genes play important role during leaf development. Collectively, it can be concluded that the highly expression of *TaEXPA1* in the young leaves may impact cell proliferation or expansin, then affect leaf development in turn.

Plenty of studies have shown that polyploidization is often accompanied by changes in genomic structure and gene expression, including loss of function, gene silencing and homoeologous gene expression diversification [2], [9], [11], [12], [35], [36]. For example, comparison of three *WLHS1* homoeologous gene sequences in wheat indicated that *WLHS1-A* has a 3479 base pair insertion, leading to the loss of gene function [10]. The expression of duplicated genes resulting from polyploidization can be partitioned between the duplicates so that only one copy is expressed and functions only in some organs, while the other copy is expressed only in other organs, indicative of subfunctionalization [37], [38]. Evolution of the *Q/q* loci in polyploid wheat resulted in the hyperfunctionalization of *5AQ*, pseudogenization of *5Bq*, and subfunctionalization of *5Dq* [39]. In present study, overexpression of *TaEXPA1-A*, *-B* and *-D* produced similar morphological changes in transgenic *Arabidopsis* plants, including increased germination and growth rate during the seedling and adult stages (Figure 3–6). These results indicated that three *TaEXPA1* homoeologs all functioned in leaf growth of transgenic *Arabidopsis* plants, although the *TaEXPA1-B* silenced in seedling leaves of wheat. Therefore, our present study provided an example of a set of homoeologous genes expression divergence in different developmental stages and organs in hexaploid wheat but functional retention in transgenic *Arabidopsis* plants. It should to be noted that, in our present study, only seed germination and seedling related traits (root and leaf) were analyzed between transgenic lines and wild-type, further analysis of other processes, such as pollination, floral opening, meristem dynamics, fruit softening, as well as in adaptive responses to submergence, GA and wounding, will provide more valuable information for the function retention of three *TaEXPA1* homoeologs.

### Overexpression of *TaEXPA1* Genes in *Arabidopsis* Leads to Increased Growth Rate

*Expansin* genes have been isolated from a variety of plant species and are a large multigene family [40], [41], [42]. It has been shown that expansins play important roles in plant development, including seed germination [23], root hair emergence [24], [43], leaf growth [25], [26], stem elongation [44], pollination [45], floral opening [46], meristem dynamics [47], fruit softening [22], as well as in adaptive responses to submergence, GA and wounding [48], and other developmental processes where cell wall loosening occurs [21]. Different members of *expansin* are expressed in various parts of plants at different developmental stages. For example, *LeEXP4* was shown to be expressed specifically in the endosperm cap tissue enclosing the radicle tip [49]. However, *LeEXP8* and *LeEXP10* were expressed in the embryo. The tissue localization and expression patterns of both *LeEXP8* and *LeEXP10* suggested that they played roles in the initial elongation stage of the radicle and seedling growth [23]. The *GmEXP1* was expressed mainly in the regions undergoing cell division and elongation in the primary and secondary roots, suggesting a role in root elongation in soybean [50]. The complexity of *expansin* gene expression is reflected in the similarly diverse expression patterns found in wheat, in which three *TaEXPA1* homoeologs were differentially expressed in different tissues and developmental stages [27]. The distinct spatial and temporal expression patterns of three *TaEXPA1* homoeologs suggested that *TaEXPA1* may play multiple roles in wheat development. In present study, we found that germination rate and primary root length of transgenic plants were faster than control plants, suggesting that *TaEXPA1* gene plays an important biological role in control of seed germination and initial elongation of the radicle. A previous study demonstrated that *AtEXP10* is expressed predominantly in young growing petioles and leaf blades, overexpression of *AtEXP10* produced larger rosettes and matured earlier as compared to the control plants, and leaf size was substantially reduced in antisense lines with suppressed *AtEXP10* expression [51]. A quantitative growth analysis by down-regulating the expression of four expansin genes (*EXPA1*, *−3*, *−5*, and *−10*) demonstrated that expansins are required for leaf growth [32]. Overexpression of three *TaEXPA1* homoeologs in *Arabidopsis* increased leaf size during the seedling stage, combined higher mRNA abundance of *TaEXPA1-A* and *TaEXPA1-D* gene in tender leaves than mature leaves, suggesting that *TaEXPA1-A* and *-D* genes may play an important biological role in leaf growth in wheat. However, transgenic study using heterologous *Arabidopsis* system only provides the function of protein from transgene in *Arabidopsis* plants. Therefore, the relationship of *TaEXPA1* genes to leaf growth in wheat is still an area to be further elucidated.

## Supporting Information

Figure S1 **Alignment of genomic and their corresponding cDNA sequences of three** ***TaEXPA1*** **homoeologous genes.** Exons and introns are showed in shaded and gray texts, respectively; ATG and TAG boxes are the start and stop codon, respectively.
(TIF)

Figure S2 **Identification of** ***TaEXPA1-A*** **,** ***TaEXPA1-B*** **and** ***TaEXPA1-D*** **gene expression in homozygous T3 transgenic** ***Arabidopsis*** **by RT-PCR.** 1 represented wide-type *Arabidopsis*; 2–4 represented overexpression *TaEXPA1-A* transgenic line of OEA-2-12, OEA-8-10, and OEA-11-12, respectively; 5–7 represented *TaEXPA1-B* transgenic line of OEB-4-6, OEB-9-9, and OEB-17-5, respectively; 8–10 represented *TaEXPA1-D* transgenic line of OED-5-3, OED-7-5, and OED-12-7, respectively.
(TIF)

Figure S3 **Cytological observation showed that the epidermal cell size of transgenic plants and wild-type did not differ significantly, while the transgenic plants rosette leaves were significantly greater than the wild-type, implying that transgenic plants were larger mainly due to an increased number of cells. Bar = 100 µm.**
(TIF)

Table S1 **The GenBank accession numbers of selected expansions.**
(DOC)

Table S2 **Gene-specific primer pairs used in PCR.**
(DOC)
