# Supplementary figures and images for: Overexpression of Three TaEXPA1 Homoeologous Genes with Distinct Expression Divergence in Hexaploid Wheat Exhibit Functional Retention in Arabidopsis
Source: PLoS One. 2013 May 16;8(5):e63667. doi: 10.1371/journal.pone.0063667 (PMC3656044; doi:10.1371/journal.pone.0063667)

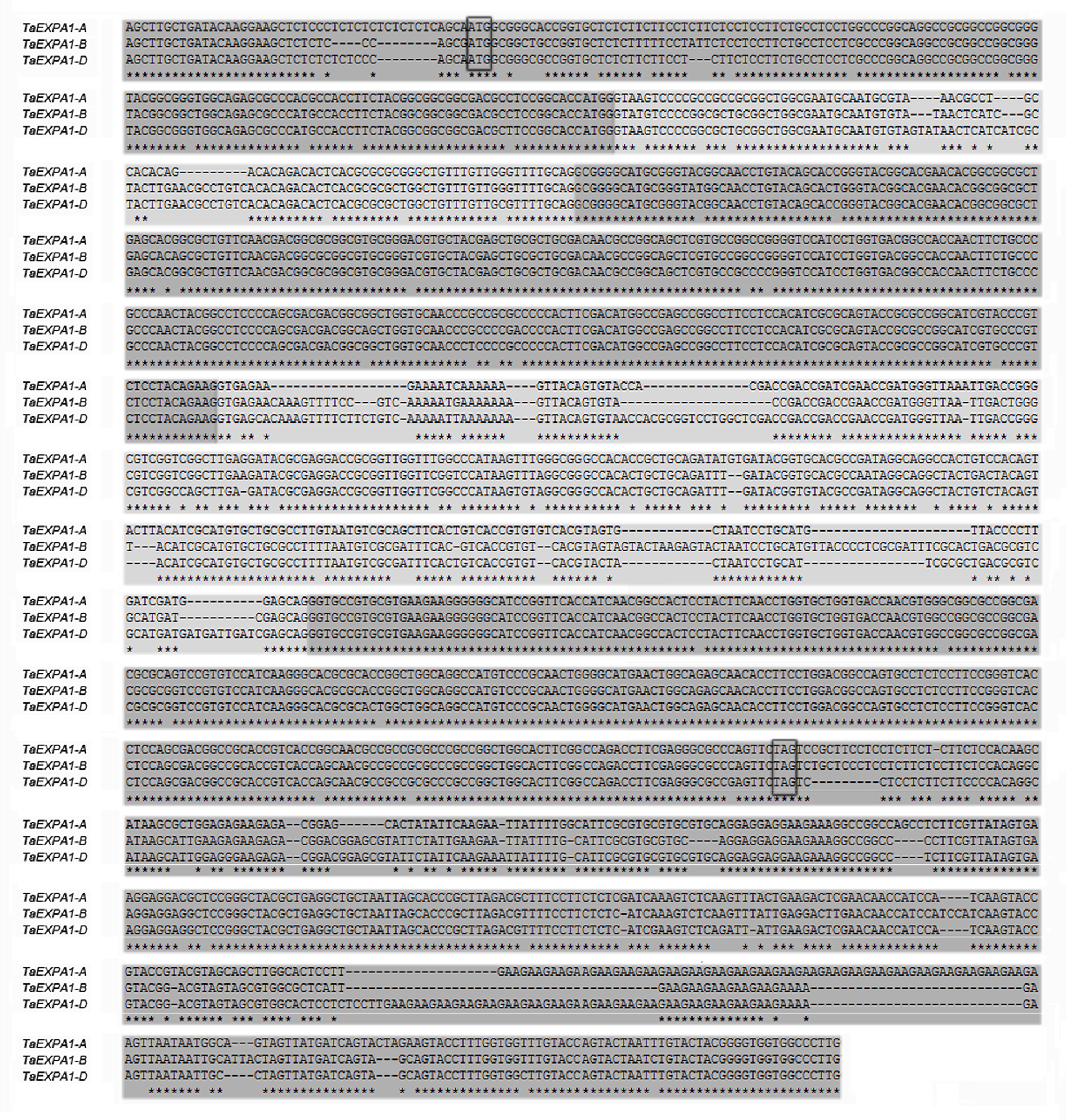

Supplement: Figure S1 — Alignment of genomic and their corresponding cDNA sequences of three TaEXPA1 homoeologous genes. Exons and introns are showed in shaded and gray texts, respectively; ATG and TAG boxes are the start and stop codon, respectively. (TIF) [file pone.0063667.s001.tif]

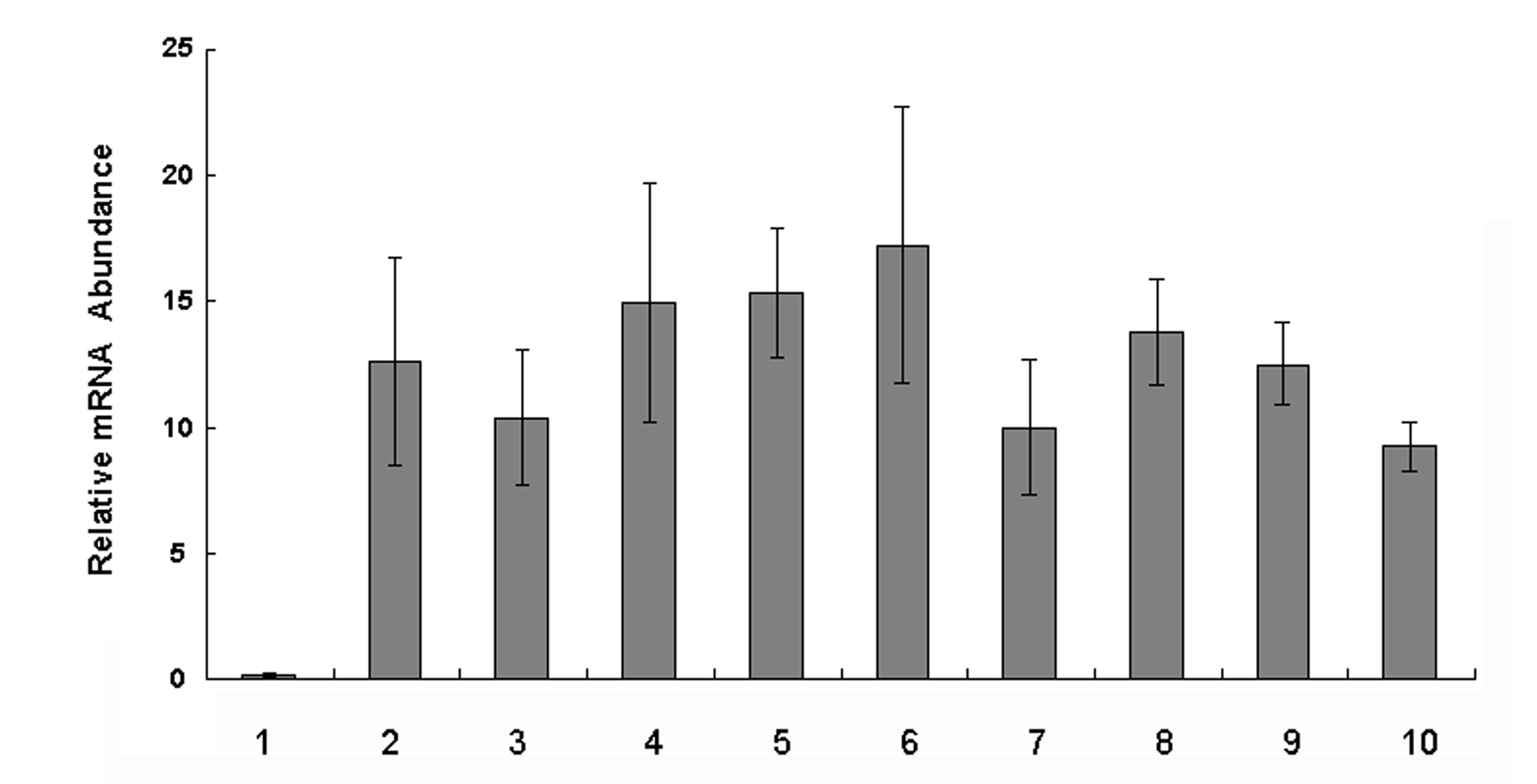

Supplement: Figure S2 — Identification of TaEXPA1-A , TaEXPA1-B and TaEXPA1-D gene expression in homozygous T3 transgenic Arabidopsis by RT-PCR. 1 represented wide-type Arabidopsis; 2–4 represented overexpression TaEXPA1-A transgenic line of OEA-2-12, OEA-8-10, and OEA-11-12, respectively; 5–7 represented TaEXPA1-B transgenic line of OEB-4-6, OEB-9-9, and OEB-17-5, respectively; 8–10 represented TaEXPA1-D transgenic line of OED-5-3, OED-7-5, and OED-12-7, respectively. (TIF) [file pone.0063667.s002.tif]

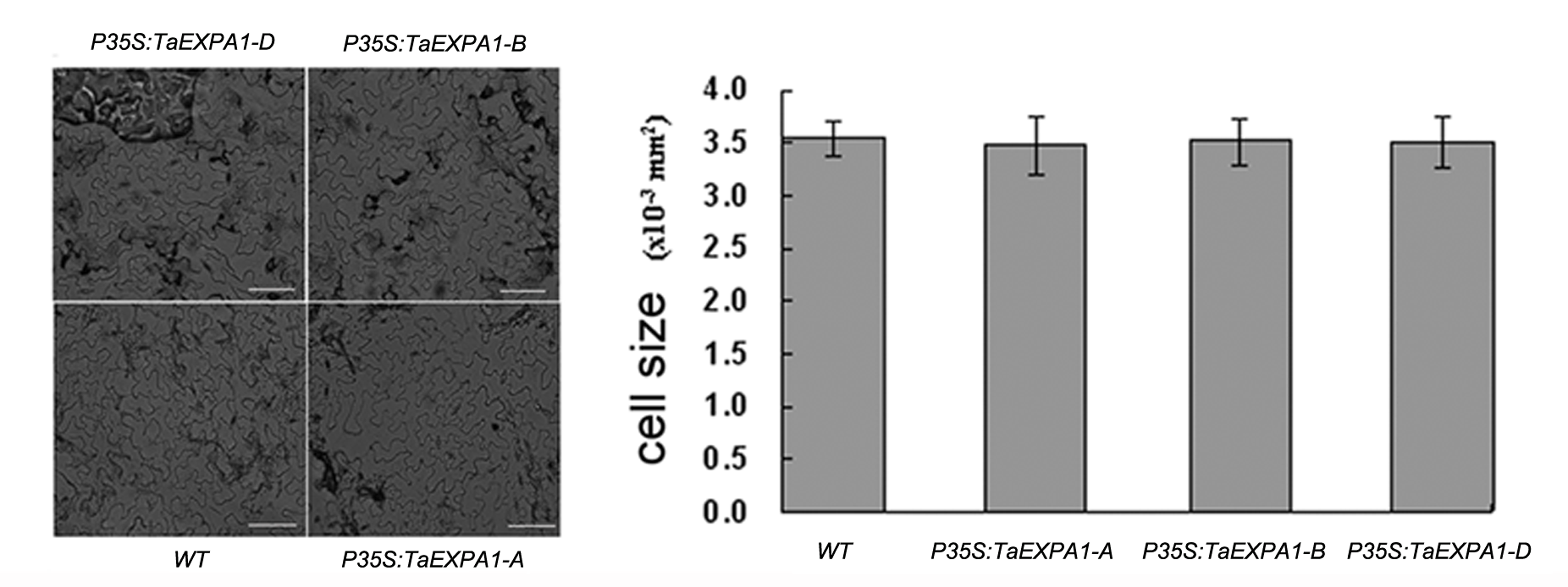

Supplement: Figure S3 — Cytological observation showed that the epidermal cell size of transgenic plants and wild-type did not differ significantly, while the transgenic plants rosette leaves were significantly greater than the wild-type, implying that transgenic plants were larger mainly due to an increased number of cells. Bar = 100 µm. (TIF) [file pone.0063667.s003.tif]
