# Supplementary material for: Overexpression of Three TaEXPA1 Homoeologous Genes with Distinct Expression Divergence in Hexaploid Wheat Exhibit Functional Retention in Arabidopsis
Source: PLoS One. 2013 May 16;8(5):e63667. doi: 10.1371/journal.pone.0063667 (PMC3656044; doi:10.1371/journal.pone.0063667)
Supplement: Table S1 — The GenBank accession numbers of selected expansions. (DOC) [file pone.0063667.s004.doc]

**Table S1.** The GenBank accession numbers of selected expansins

| **Name** | **Chr #** | **Locus** | **Name** | **Chr #** | **Locus** |
| --- | --- | --- | --- | --- | --- |
| AtEXPA1 | 1 | At1g69530 | OsEXPA1 | 4 | Os04g0228400 |
| AtEXPA2 | 5 | At5g05290 | OsEXPA2 | 1 | Os01g0823100 |
| AtEXPA3 | 2 | At2g37640 | OsEXPA3 | 5 | Os05g0276500 |
| AtEXPA4 | 2 | At2g39700 | OsEXPA4 | 5 | Os05g0477600 |
| AtEXPA5 | 3 | At3g29030 | OsEXPA5 | 2 | Os02g0744200 |
| AtEXPA6 | 2 | At2g28950 | OsEXPA6 | 3 | Os03g0336400 |
| AtEXPA7 | 1 | At1g12560 | OsEXPA7 | 3 | Os03g0822000 |
| AtEXPA8 | 2 | At2g40610 | OsEXPA8 | 1 | Os01g0248900 |
| AtEXPA9 | 5 | At5g02260 | OsEXPA9 | 1 | Os01g0249100 |
| AtEXPA10 | 1 | At1g26770 | OsEXPA10 | 4 | Os04g0583500 |
| AtEXPA11 | 1 | At1g20190 | OsEXPA11 | 1 | Os01g0274500 |
| AtEXPA12 | 3 | At3g15370 | OsEXPA12 | 3 | Os03g0155300 |
| AtEXPA13 | 3 | At3g03220 | OsEXPA13 | 2 | Os02g0267200 |
| AtEXPA14 | 5 | At5g56320 | OsEXPA14 | 2 | Os02g0267700 |
| AtEXPA15 | 2 | At2g03090 | OsEXPA15 | 3 | Os03g0155600 |
| AtEXPA16 | 3 | At3g55500 | OsEXPA16 | 6 | Os06g0621900 |
| AtEXPA17 | 4 | At4g01630 | OsEXPA17 | 6 | Os06g0108600 |
| AtEXPA18 | 1 | At1g62980 | OsEXPA18 | 3 | Os03g0155900 |
| AtEXPA19 | 3 | At3g29365 | OsEXPA19 | 3 | Os03g0156000 |
| AtEXPA20 | 4 | AT4g38210 | OsEXPA20 | 3 | Os03g0156300 |
| AtEXPA21 | 5 | AT5g39260 | OsEXPA21 | 3 | Os03g0377100 |
| AtEXPA22 | 5 | AT5g39270 | OsEXPA22 | 2 | Os02g0268600 |
| AtEXPA23 | 5 | AT5g39280 | OsEXPA23 | 2 | Os02g0268050 |
| AtEXPA24 | 5 | AT5g39310 | OsEXPA24 | 2 | Os02g0267900 |
| AtEXPA25 | 5 | AT5g39300 | OsEXPA25 | 3 | Os03g0155500 |
| AtEXPA26 | 5 | AT5g39290 | OsEXPA26 | 12 | Os12g0546800 |
|  |  |  | OsEXPA27 | 10 | Os10g0439100 |
| **Name** | **Chr #** | **Accession Number** | OsEXPA28 | 10 | Os10g0439200 |
| TaEXPA1-A | 1A | AY910581.1 | OsEXPA29 | 6 | Os06g0718100 |
| TaEXPA1-B | 1B | AY485121.3 | OsEXPA30 | 10 | Os10g0535900 |
| TaEXPA1-D | 1D | AY910580.1 | OsEXPA31 | 3 | Os03g0428700 |
|  |  |  | OsEXPA32 | 8 | Os08g0561900 |
|  |  |  | OsEXPA33 | 5 | Os05g0277000 |
