# Supplementary material for: Overexpression of Three TaEXPA1 Homoeologous Genes with Distinct Expression Divergence in Hexaploid Wheat Exhibit Functional Retention in Arabidopsis
Source: PLoS One. 2013 May 16;8(5):e63667. doi: 10.1371/journal.pone.0063667 (PMC3656044; doi:10.1371/journal.pone.0063667)
Supplement: Table S2 — Gene-specific primer pairs used in PCR. (DOC) [file pone.0063667.s005.doc]

**Table S2. Gene-specific primer pairs used in PCR.**

| **Name** | **Sequence (5´-3´)** |  |
| --- | --- | --- |
| TaEXPA1-ABL | GCTACGCTGAGGCTGCTAAT | Quantitative PCR analysis |
| TaEXPA1-AR | CTAGTACTGATCATAACTAC |
| TaEXPA1-BR | CTACTGATCATAACTAGTAA |
| TaEXPA1-DL | GCGCCGAGTTCTAGTCCT |
| TaEXPA1-DR | CAAATTAGTACTGGTACAAG |
| β-actin-L | GGAATCCATGAGACCACCTAC |
| β-actin-R | GACCCAGACAACTCGCAAC |
| TaEXPA1-L | CAAAAAAGCAGGCTCAGCTTGCTGATACAAGGAAGCTC | Binary Constructs |
| TaEXPA1-R | CAAGAAAGCTGGGTCAGGGCCACCACCCCGTAGTAC |
